# Quantum Walk on the Generalized Birkhoff Polytope Graph

**DOI:** 10.3390/e23101239

**Published:** 2021-09-23

**Authors:** Rafael Cação, Lucas Cortez, Ismael de Farias, Ernee Kozyreff, Jalil Khatibi Moqadam, Renato Portugal

**Affiliations:** 1 Department of Industrial, Manufacturing & Systems Engineering, Texas Tech University, Lubbock, TX 79430, USA; rafael.cacao@ttu.edu (R.C.); lucas.cortez@ttu.edu (L.C.); 2 Campus of Itapeva, Universidade Estadual Paulista (Unesp), Itapeva 18409-010, SP, Brazil; ernee.kozyreff@unesp.br; 3 National Laboratory of Scientific Computing (LNCC), Petrópolis 25651-076, RJ, Brazil; jalilkhm@lncc.br (J.K.M.); portugal@lncc.br (R.P.)

**Keywords:** quantum walk, transportation problem, generalized Birkhoff polytope, counting, sampling

## Abstract

We study discrete-time quantum walks on generalized Birkhoff polytope graphs (GBPGs), which arise in the solution-set to certain transportation linear programming problems (TLPs). It is known that quantum walks mix at most quadratically faster than random walks on cycles, two-dimensional lattices, hypercubes, and bounded-degree graphs. In contrast, our numerical results show that it is possible to achieve a greater than quadratic quantum speedup for the mixing time on a subclass of GBPG (TLP with two consumers and *m* suppliers). We analyze two types of initial states. If the walker starts on a single node, the quantum mixing time does not depend on *m*, even though the graph diameter increases with it. To the best of our knowledge, this is the first example of its kind. If the walker is initially spread over a maximal clique, the quantum mixing time is O(m/ϵ), where *ϵ* is the threshold used in the mixing times. This result is better than the classical mixing time, which is $O(m^{1.5}/\epsilon )$.

## 1. Introduction

The transportation linear programming problem (TLP) is to find the cheapest way to send a commodity from suppliers directly to consumers to satisfy the demands of the consumers, restricted to the capacities of the suppliers. It is a fundamental problem in operations research, management science, and the economics of scarce resources. Besides being important on its own, it appears as a subproblem in other optimization models [1]. TLP can be solved efficiently, for example, by the Network Simplex algorithm [2].

The solution-set to TLP, the *transportation polytope*, induces a graph, the *transportation polytope graph* (TPG; also called the transportation polytope skeleton), whose nodes are a special class of solutions to TLP, some of which are optimal solutions. For this reason, counting and sampling the vertices of TPG is of interest, and so is the random walk (RW) on TPG [3]. However, the literature about it is scarce; we are not aware of any study of RW on TPG other than that in [4].

Along the same line, quantum walks (QWs) on TPG are of interest, because they are the quantum counterpart of RW and they promise better sampling [5]. We are not aware of any study of QW on TPG.

Quantum computing has gained much attention nowadays, after quantum supremacy has been established by Google [6] and recent Chinese experiments [7]. QW [8] can be efficiently implemented in quantum computers [9] and the main barrier to deliver results is the accumulation of noise during the computation. The term NISQ computers [10] has been coined by Preskill to classify the quantum computers that are used nowadays and will be in use in the near future.

In this paper, we study the discrete-time QW on a particular family of TPG, the generalized Birkhoff polytope graph [11], or GBPG for short, with the focus on the quantum mixing time. To define mixing times, we need the notion of limiting probability distribution. In the classical case, the limiting distribution is the stationary probability distribution of a Markov chain, which is always reached on ergodic Markov chains, in the limit of infinitely many steps. If the graph on which RW takes place is regular, the classical limiting probability distribution is uniform [12].

In the quantum case, the probability distribution of QW does not converge to a stationary distribution, and it is necessary to define the notion of *average* probability distribution. The average distribution always converges to a quantum limiting distribution, which depends on the initial state of QW. The quantum mixing time is the number of steps required to reach or to be ϵ-close to the limiting distribution.

We analyze coined QW and RW on a subclass of GBPG (TLP with two consumers and *m* sources). We do so by simulating both quantum and classical walks numerically to then determine, through curve fitting, how the mixing time scales with *m* in both cases. In the quantum case, this also means finding the limiting distribution.

Our numerical results show that the classical mixing time is $O(m^{1.5}/\epsilon )$. On the other hand, the quantum mixing time is O(m/ϵ) if the initial state is localized on a maximal clique (A clique in a graph is a subset of the set of nodes, such that any two nodes in the subset are adjacent. A clique is maximal if it is not a proper subset of a clique.) of GBPG. If the initial state is localized on an arbitrary node, the quantum mixing time is O(1/ϵ), which, to the best of our knowledge, is the first example where QW delivers a speedup over RW for the mixing time that is greater than quadratic. In addition, in this case, the quantum mixing time, as a function of *m*, does not increase, even though, as we show later, the graph diameter increases with it. To the best of our knowledge, this is also the first example of its kind.

We give asymptotic results by extrapolating our numerical results, based on the assumption that the overall structure of GBPG remains the same even for large values of *m*. Using the amplification technique described in [5], the dependence of the mixing time on ϵ can be improved without changing its dependence on *m*.

The paper is organized as follows. In Section 2, we introduce concepts, terminology, and results on linear programming, polytopes, and polytope graphs, which we will use in the remainder of the paper. In Section 3, we describe TLP, and we introduce the transportation polytope and the generalized Birkhoff polytope. We then define GBPG and give some of its properties. In Section 4, we define the mixing time of RW on GBPG and we review discrete-time QW. We then define the average probability distribution and the mixing time of QW on GBPG. In Section 5, we describe our numerical simulation of QW and RW on GBPG, and our results on the limiting probability distribution and the quantum and classical mixing times. In Section 6, we present our conclusions and directions for further research.

## 2. Linear Programming Problems, Polytopes, and Polytope Graphs

In this section, we introduce concepts, terminology, and results on linear programming, polytopes, and polytope graphs, which we will use in the remainder of the paper.

The linear programming problem (LP) is

minimizez=c1x1+…+cdxds.t.(1) ∑j=1daijxj=bi,i=1,…,r(2) x1,…,xd≥0.

We call *z* the objective function of LP. The set

$$P=\left\{x\in {ℜ}^{d}:\left(1\right)\mathrm{and}\left(2\right)\mathrm{hold}\right\}$$

is the *solution-set* of LP. When P≠∅, we say that LP is *feasible*. A point x∈P is a *feasible solution* to LP. A feasible solution to LP that minimizes *z* (when one exists) is an *optimal solution*.

Set P is a *polyhedron*. In this paper, we will deal exclusively with bounded polyhedra, so we will assume for the remainder of this section that P is bounded. We call a bounded polyhedron a *polytope*.

The *0-dimensional faces* of P correspond to a special class of solutions to LP, called *basic feasible solutions* (BFS’s). The importance of BFS is that (1) a point in a polytope is a convex combination of its 0-dimenisonal faces, so a solution to LP is a convex combination of its BFS’s, and (2) when LP has an optimal solution, at least one of them is a BFS, and any optimal solution to LP is a convex combination of optimal BFS’s. This result is known as the Fundamental Theorem of Linear Programming [13]. It means that the search for an optimal solution to LP can be restricted to searching an optimal BFS. This is the basis of the celebrated Simplex algorithm [13].

When polytope P is nonempty, we define its graph (also called skeleton) as follows. The nodes of the graph are the 0-dimensional faces of the polytope (the BFS’s of LP), and two nodes are adjacent when they are joined by a 1-dimensional face of P. We note that when P≠∅, LP always has an optimal solution.

Different formulations of LP will give different solution-sets, and in the case of nonempty polytopes, different polytope graphs, possibly with different properties. An important problem in optimization (of which LP is a special case) is to determine, for different classes of problems, which formulation leads to a more efficient solution approach or is more convenient to investigate within a given context.

## 3. TLP, the Generalized Birkhoff Polytope, and GBPG

In this section, we describe TLP, and we introduce the transportation polytope and the generalized Birkhoff polytope. We then define GBPG and give some of its properties.

Let *m* be the number of sources and *n* the number of consumers. We denote the sets of sources and consumers as $M=\left\{1,\ldots ,m\right\}$ and $N=\left\{1,\ldots ,n\right\}$, respectively. Let $G=\left(M,N;A\right)$ be a complete directed bipartite network, with node sets M and N, and arc set A=M×N (for an introduction to graph theory and network flows, see [14]). To simplify notation, arc $\left(i,j\right)\in A$ is denoted as ij.

Let $s_{i}>0$ be the capacity of source i∈M and $d_{j}>0$ the demand of consumer j∈N. Let $c_{ij}\geq 0$ be the cost of shipping one unit of the commodity from *i* to *j* (if arc ij is not present in a particular instance, we account for this in our model by taking $c_{ij}=\infty$). TLP is

minimizez=∑ij∈Acijxijs.t.(3)∑j∈Nxij=si,i∈M(4)∑i∈Mxij=dj,j∈N(5) xij≥0,ij∈A.

Because TLP is trivial when m=1 or n=1, we assume that m,n≥2. We assume without loss of generality that

$$\sum_{i\in M}s_{i}=\sum_{j\in N}d_{j},$$

in which case TLP is feasible. The solution-set to TLP

$$P=\left\{x\in {ℜ}^{mn}:\left(3\right),\left(4\right),\mathrm{and}\left(5\right)\mathrm{hold}\right\}$$

is a polytope, and TLP has an optimal solution.

Two particularly important special cases of TLP are the generalized Birkhoff linear programming problem (denoted TLPGB) and the assignment (also called Birkhoff) linear programming problem. TLPGB is obtained by taking

(6) $$s_{1}=\ldots =s_{m}=n\;\mathrm{and}\;d_{1}=\ldots =d_{n}=m.$$

The assignment linear programming problem is TLPGB with m=n.

As it is formulated, the graph of the solution-set to TLPGB is not regular in general. However, it is possible to formulate TLPGB in a way that the graph of its solution-set is regular. This is accomplished by using *perturbation* to eliminate *degeneracy* in BFS, see in [15]. Because it is more convenient to analyze classical and quantum mixing times on a regular graph, we will adopt this formulation. The feasible-set of TLPGB formulated without degeneracy will be called generalized Birkhoff polytope and denoted $P_{GB}$. Its graph will be called generalized Birkhoff polytope graph (GBPG). The degree of GBPG is $\left(m-1\right)\left(n-1\right)$. GBPG has the highest number of nodes among all TPGs [16].

TLP was first studied by Hitchcock [17] and Koopmans [18]. Since then, a flood of research followed, see, for example, in [19,20,21] and references therein to name just a few. An active area of research is the properties of the transportation polytope, and the generalized Birkhoff polytope in particular [11,15,16,22,23,24].

## 4. Classical and Quantum Walk, and Their Mixing Times

In this section, we define the classical mixing time of RW on GBPG and we review discrete-time QW. We then define average probability distribution and the quantum mixing time of QW on GBPG.

### 4.1. RW on GBPG

RW on GBPG is described by a sequence of probability distributions $\left\{p(t):t=0,1,2,\ldots \right\}$ on the nodes of GBPG, and a transition matrix *M*, obtained from the adjacency matrix of GBPG, so that p(t+1)=Mp(t). For n=2 and m=4,…,15, the Markov chain on GBPG is ergodic [12], and we conjecture that this is true for n=2 and m≥4. In any case, for the instances we simulate in this paper, p(t) converges to a limit distribution π, regardless of the initial distribution p(0). As GBPG is regular, π is the uniform distribution.

The classical mixing time is defined to be

(7) $$\tau_{\epsilon }=\min\left\{T\;|\;\forall t\geq T,\;∥p(t)-\pi ∥\leq \epsilon \right\},$$

where the threshold ϵ is a positive number and ∥p(t)−π∥ is the total variation distance of p(t) and π, given by

(8) $$∥p\left(t\right)-\pi ∥=\frac{1}{2}\sum_{v\in V}\left|p_{v}\left(t\right)-\pi_{v}\right|.$$

In other words, the mixing time is the minimum number of steps required to the average distribution to be ϵ-close to the limiting distribution π. The mixing time is an integer number. However, note that we obtain the mixing time using curve fitting methods, which may result in non-integer values.

Despite its importance, very little is known about RW on TPG, GBPG, or the assignment polytope graph, and consequently little is known about enumerating or sampling their nodes. Dyer [25] showed that it is #P to count the number of nodes of TPG exactly, even when the number of sources is fixed at 2. Cryan et al. [4] gave a random walk on TPG that mixes in time $n^{O(m^{2})}$. Therefore, the random walk mixes rapidly when *m* is fixed. Pak [24] showed that RW on the assignment polytope graph mixes in only two steps in the limit n→∞. We are not aware of any results specifically for RW on GBPG.

It is possible to extend the concepts of this and the previous section to define graphs of other polytopes and RW on them. Besides the aforementioned papers on RW for TPG and special cases, the only papers on RW on other polytope graphs arising in optimization that we are aware of are those in [26,27]. We are not aware of any work on the quantum walk on the graph of an optimization polytope.

### 4.2. QW on GBPG

#### 4.2.1. QW

To define a discrete-time coined quantum walk on GBPG, which is a regular graph with degree D=(m−1)(n−1), we employ a Hilbert space $H^{D}⊗H^{N}$ with computational basis $\left\{|a,v\rangle :1\leq a\leq D,\;v\in V\right\}$, where *V* is the node set of GBPG and |V|=N. A generic state of the quantum walk after *t* steps is

$$|\psi (t)\rangle =\sum_{a=1}^{D}\sum_{v\in V}\psi_{a,v}\left(t\right)|a,v\rangle ,$$

where $\psi_{a,v}\left(t\right)$ are the amplitudes, which must be normalized, i.e., $\sum_{a,v}{\left|\psi_{a,v}\left(t\right)\right|}^{2}=1$ ∀t≥0. The dynamics of the quantum walk is given by

$$|\psi (t)\rangle =U^{t}|\psi (0)\rangle ,$$

where *U* is the evolution operator defined as

U=S·(C⊗I).

The coin operator *C* is a *D*-dimensional unitary operator, which is usually the Grover coin with entries $C_{ij}=2/D-\delta_{ij}$, where $\delta_{ij}$ is the Kronecker delta. Operator *S* is the flip-flop shift operator, whose action on the element $|a,v\rangle$ of the computational basis is $S|a,v\rangle =|a^{\prime },w\rangle$, where *w* is a node in the neighborhood of *v*, *a* is the label of the arc with tail *v* and head *w*, and $a^{\prime }$ is the label of the arc with tail *w* and head *v*. The initial state $|\psi (0)\rangle$ is usually taken localized on a node *v* with the coin state uniformly superposed, that is, $|\psi (0)\rangle =|u\rangle ⊗|v\rangle$, where $|u\rangle =\sum_{a=1}^{D}|a\rangle /\sqrt{D}$. In this paper, we also consider initial states distributed over a maximal clique *K*, in this case

$$|\psi (0)\rangle =\frac{1}{\sqrt{\left|K\right|}}|u\rangle \sum_{v\in K}|v\rangle .$$

The dynamics of the quantum walk leads to a probability distribution $p_{v}\left(t\right)$ over the node set v∈V, defined as

(9) $$p_{v}\left(t\right)=\sum_{a=1}^{D}{\left|\langle a,v|\psi (t)\rangle \right|}^{2},$$

which means that if one measures the position of the walker after *t* steps in the computational basis, the probability of finding the walker at node *v* is $p_{v}\left(t\right)$. In the quantum regime, the probability distribution does not converge in the limit where the number of steps *t* goes to infinity because in the unitary dynamics the state of the walk is quasi-periodic, in the sense that there is an infinite number of time-steps $t_{1},t_{2},\ldots$ such that the states $|\psi (t_{1})\rangle$, $|\psi (t_{2})\rangle$, … are ϵ-close to the initial state $|\psi (0)\rangle$∀ϵ>0.

#### 4.2.2. Limiting Distribution and Mixing Time

The average probability distribution at time *T* is defined as

(10) $${\bar{p}}_{v}\left(T\right)=\frac{1}{T}\sum_{t=0}^{T-1}p_{v}\left(t\right),$$

see in [5], which can be experimentally reproduced by repeatedly running the quantum walk *t* steps, where *t* is randomly selected in the range [0,T−1], and then performing a position measurement. As the notion of average distribution incorporates measurements, ${\bar{p}}_{v}\left(T\right)$ evolves stochastically as a function of *T*.

Using Equation (9), we obtain

$${\bar{p}}_{v}\left(T\right)=\frac{1}{T}\sum_{t=0}^{T-1}\sum_{a=1}^{D}{\left|\langle a,v|\psi (t)\rangle \right|}^{2}.$$

The time evolution of ${\bar{p}}_{v}\left(T\right)$ converges to a limiting distribution, which is defined as

(11) $$\pi \left(v\right)=\lim_{T\to \infty }{\bar{p}}_{v}\left(T\right).$$

Ahoronov et al. [5] give analytical expressions for π(v) in terms of a normalized eigenbasis $|\lambda_{a,v}\rangle$ of the evolution operator. In the non-degenerate case, if $|\psi (0)\rangle =\sum_{a,v}c_{a,v}|\lambda_{a,v}\rangle$, then the limiting distribution is

(12) $$\pi \left(v\right)=\sum_{a,b=1}^{D}\sum_{v^{\prime }\in V}{\left|c_{a,v^{\prime }}\langle b,v|\lambda_{a,v^{\prime }}\;\rangle \right|}^{2}.$$

Note that usually the limiting distribution depends on the initial condition. Ahoronov et al. [5] show that quantum walks on Cayley graphs of Abelian groups have uniform limiting distribution in the non-degenerate case. In this case, the limiting distribution does not depend on the initial condition. The limiting distributions for the graphs analyzed in this paper depend on the initial conditions.

As the average probability distribution always converges to a limiting distribution π, the quantum mixing time is defined as

(13) $$\tau_{\epsilon }=\min\left\{T\;|\;\forall t\geq T,\;∥\bar{p}\left(t\right)-\pi ∥\leq \epsilon \right\},$$

where ϵ is a positive number and $∥\bar{p}\left(t\right)-\pi ∥$ is the total variation distance of $\bar{p}\left(t\right)$ and π is given by Equation (8).

There is another time measure, called instantaneous mixing time, which captures the first instant that the instantaneous probability distribution is ϵ-close to some reference probability distribution μ. The instantaneous mixing time is defined as

(14) $$I_{\epsilon }=\min\left\{t\;|\;∥p(t)-\mu ∥\leq \epsilon \right\},$$

where p(t) is the quantum probability distribution at time *t*. One can replace μ by the uniform probability probability distribution to analyze the time it takes to obtain an uniform sampling.

In the literature, one finds papers analyzing the quantum mixing time on cycles [5], Cayley graphs [5], hypercubes [28,29], two-dimensional lattices [30], and complete graphs [31]. Upper bounds for the quantum mixing time were obtained in [32,33]. Apers et al. [34] discuss the simulation of the quantum mixing time by classical Markov chains with added memory, and concludes that quantum walk speedups are not necessarily diagnostic of quantum effects. The Ph.D. thesis [35] has an interesting description on how Markov chain lifting can simulate fast quantum mixing times.

## 5. Simulation and Numerical Results for RW and QW on GBPG

In this section, we give details of our simulation of RW and QW on GBPG with n=2 and m=2,…,15. We first describe how these 14 instances were generated. Then, we explain how we obtained their classical and quantum mixing times, and how they scale with *m*.

### 5.1. Computational Platform and Instance Generation

The bulk of our computation was performed through the Texas Tech High-Performance Computing Center. We used the Nocona partition, with an AMD EPYC^TM^ 7702 benchmarked at 804 TFLOPS using 4 GB of RAM per node, running Linux CentOS version 8.1. The code used to calculate average limiting distributions and mixing times ran on Python 3.8.5.

Classical random walks were simulated on GBPG with n=2 consumers and m=2,…,15 suppliers, and quantum random walks were simulated on GBPG with n=2 consumers and m=2,…,11 suppliers. We used perturbation [15] to generate regular instances of TLPGB as follows. For all i∈M, the values of $s_{i}$ were decreased by $\frac{1}{2m}$, and the value of $d_{2}$ was decreased by $\frac{1}{2}$. The software PORTA [36] was used to compute all vertices of $P_{GB}$, and the adjacency matrix for GBPG was built by comparing every pair of vertices of $P_{GB}$. They are adjacent in GBPG when exactly m+n−2 variables have positive entries in both vertices. Table 1 displays, for each instance of GBPG, the number of suppliers (*m*), the number of nodes

N=mm−1(m−1)/2,

its diameter (diam.), and spectral gap $\left(1-\lambda_{1}\right)$, where $\lambda_{1}$ is the second largest eigenvalue of the stochastic transition matrix of the graph.

### 5.2. Classical Mixing Time

As GBPG is regular, the limiting probability distribution is uniform [12]. Determining the classical mixing time, employs Equations (7) and (8) by substituting the uniform probability distribution for $\pi_{v}$.

**Figure 1:**
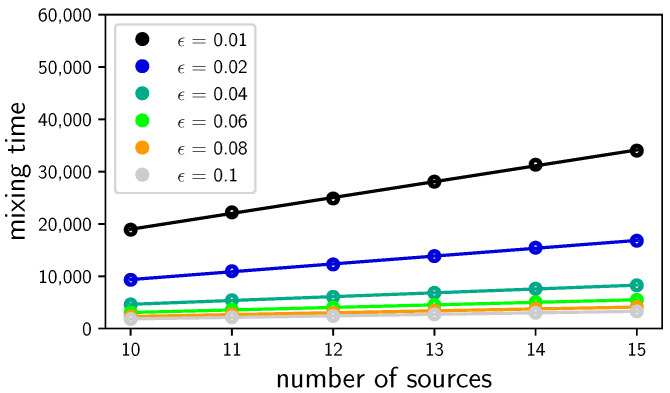
Classical mixing time as a function of the number of sources *m* for ϵ from 0.01 to 0.1.

Figure 1 shows the mixing time as a function of the number of sources *m* for ϵ between 0.01 and 0.1. When we re-scale the mixing time $\tau_{\epsilon }$ to $\epsilon \tau_{\epsilon }$, all straight lines merge into one straight line, showing that $\tau_{\epsilon }$ scales as 1/ϵ for a fixed *m*. Using the numerical data of Figure 1, we conclude that $\tau_{\epsilon }=O\left(m^{1.5}/\epsilon \right)$. This is an example of a *rapidly mixing* Markov chain because $\tau_{\epsilon }$ is polylogarithmic on the number of nodes.

As the diameter of the Birkhoff polytope is O(m), our result for the classical mixing time is consistent with the lower bound $\tau_{\epsilon }\geq \mathrm{diam}/2$ described in [37] for any threshold ϵ. Our result is also consistent with the upper bound [38]

$$\tau_{\epsilon }\leq \frac{\ln N-\ln\epsilon }{1-\lambda_{1}},$$

which is equivalent to $\tau_{\epsilon }=O\left(m^{2}\right)$ for a fixed ϵ.

### 5.3. Limiting Probability Distribution (Quantum Case)

**Figure 2:**
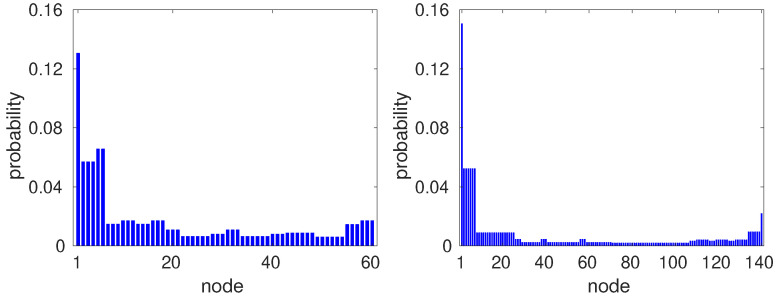
Limiting distribution $\pi_{v}$ of a quantum walk on GBPG for m=6 (left panel) and m=7 (right panel), and initial state (15) localized on node 1.

In order to calculate the quantum mixing time, we need to find the limiting distribution given by Equation (11). In numerical calculations, we usually determine the average probability distribution ${\bar{p}}_{v}\left(T\right)$ for a large number of steps *T* so that ${\bar{p}}_{v}\left(T\right)$ is close enough to the limiting distribution $\pi_{v}$. In our numerical simulations, we used $T=10^{7}$ steps. Figure 2 shows the average probability distribution ${\bar{p}}_{v}\left(10^{7}\right)$ of a quantum walk on GBPG for m=6 (left panel) and m=7 (right panel). The initial state is localized on the node with label 1, uniformly distributed over the coin values, that is,

(15) $$|\psi (0)\rangle =\frac{1}{\sqrt{m-1}}\sum_{a=1}^{m-1}|a,1\rangle .$$

The limiting distribution for m=6 (m=7) represents the class of limiting distributions when *m* is even (odd). When the initial state is localized on a node with label different from 1, the limiting distribution is a permutation of the bars of the plots of Figure 2. Note that two distinct nodes $v_{1}$ and $v_{2}$ that have the same distance to the initial position may have different probabilities $\pi_{v_{1}}\neq \pi_{v_{2}}$.

Now, we use the symmetries of GBPG to obtain a limiting distribution whose value $\pi_{v}$ at node *v* is uniquely determined as a function of the distance of *v* to the initial position of the walker. The instances of the GBPG that we are using have the following properties: (1) if *m* is odd, all maximal cliques have size (m+1)/2; (2) if *m* is even, the maximal cliques have size m/2 or (m/2+1); (3) two maximal cliques have no common arcs; and (4) any node is a common vertex of exactly two maximal cliques. We conjecture that these properties hold for arbitrary *m*. In order to obtain a wave function that spreads symmetrically, the initial state is a uniform superposition of one maximal clique. Suppose that the labels of the nodes of this maximal clique are $1,\ldots ,\left\lceil \left(m+1\right)/2\right\rceil$. The initial state is

(16) $$|\psi (0)\rangle =\frac{\sqrt{2}}{\sqrt{c}}\sum_{a=1}^{m-1}\sum_{v=1}^{\left\lceil \frac{m+1}{2}\right\rceil }|a,v\rangle ,$$

where $c=m^{2}-1$ if *m* is odd and c=(m+2)(m−1) if *m* is even.

**Figure 3:**
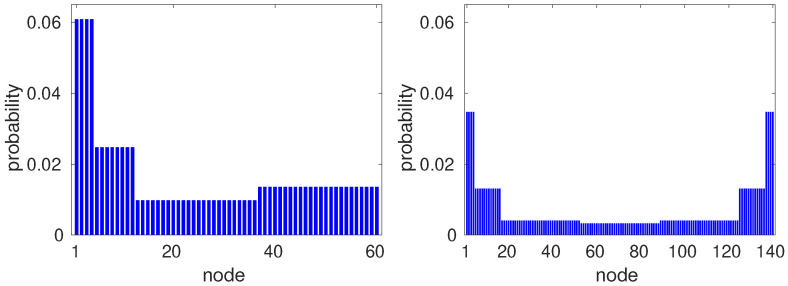
Limiting distribution $\pi_{v}$ of a quantum walk on GBPG for m=6 (left panel) and m=7 (right panel), using an initial state distributed on the clique {1,2,3,4}.

Figure 3 shows the limiting probability distribution of a quantum walk on GBPG for m=6 (left panel) and m=7 (right panel) using initial state (16). Note that for odd *m*, the limiting distribution is symmetric. In these distributions, if two nodes have the same distance to the initial clique, then they have the same probability. The reverse is not necessarily true, for instance, in the right panel of Figure 3, the probability at vertices 137, 138, 139, and 140, which is the maximal clique most distant from the initial clique, is equal to the probability at the nodes of the initial clique.

### 5.4. Quantum Mixing Time

**Figure 4:**
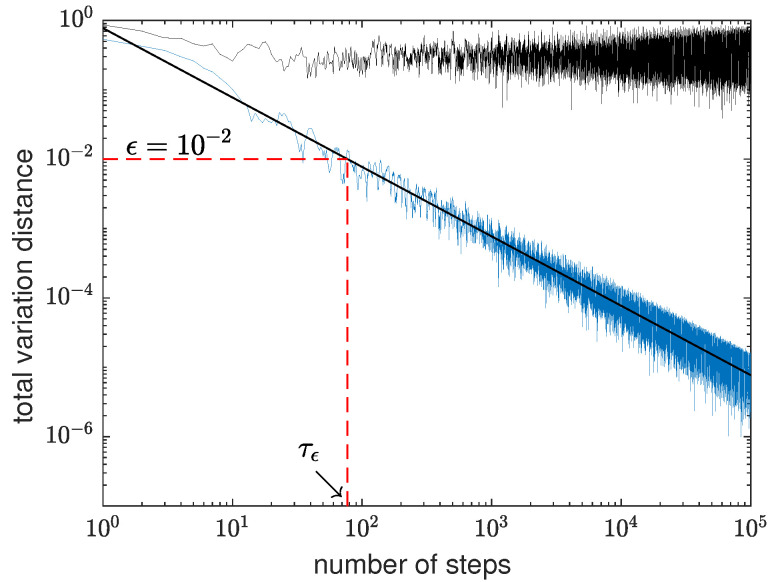
Log-log plot of the total variation distance between the instantaneous and limiting distributions ∥p(t)−π∥ (upper black curve), and between the average and limiting distributions $∥\bar{p}\left(t\right)-\pi ∥$ (lower blue curve) as a function of time steps for GBPG for m=7. The equation of the straight line is $0.7761/t^{1.0007}$.

A typical plot of the total variation distance $∥\bar{p}\left(t\right)-\pi ∥$ as a function of the number of time steps is depicted in Figure 4. In this plot, the number of sources is m=7, and the equation of the fitted straight line is $0.7761/t^{1.0007}$. The approximate limiting distribution is obtained from ${\bar{p}}_{v}\left(10^{7}\right)$ and the number of steps runs until $10^{5}$, which is much smaller than $10^{7}$. The plot shows that the behavior of $∥\bar{p}\left(t\right)-\pi ∥$ as a function of *t* is approximately f(t)/t, where f(t) is an oscillatory function with high frequency, short wavelength, and small amplitude. This behavior is ubiquitous [8,29,30,39]. Figure 4 also shows how to calculate the mixing time $\tau_{\epsilon }$ for a given threshold ϵ: $\tau_{\epsilon }$ is the number of steps (as a real number here) such that the total variation distance is equal to ϵ. This calculation method implies that the quantum mixing time we are obtaining is an average value close to the real mixing time defined in (13).

Figure 4 also depicts the total variation distance ∥p(t)−π∥ between the instantaneous distribution and the average limiting distribution, which is used to calculate the instantaneous mixing time $I_{\epsilon }$. The plot shows that the total variation distance oscillates around one-eighth with an increasing amplitude as a function of the number of steps.

**Figure 5:**
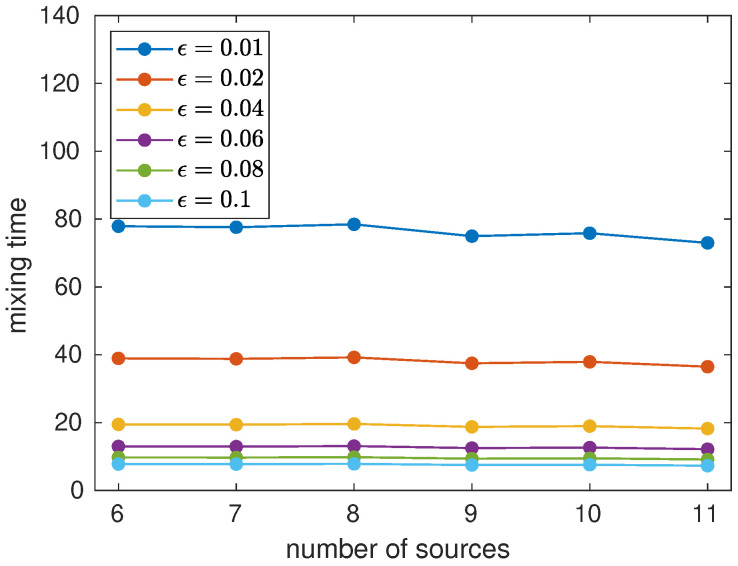
Quantum mixing time as a function of the number of sources *m* for ϵ from 0.01 to 0.1.

Figure 5 depicts the quantum mixing time as a function of the number of sources *m* for ϵ from 0.01 to 0.1. To calculate the mixing time, we have used the procedure described in Figure 4. We have selected an initial state localized on node 1 and we have used the limiting distributions of Figure 2. The numerical data shows that the mixing time does not increase when *m* increases, in fact, there is a downward tendency for m>8. If we re-scale $\tau_{\epsilon }$ as $\epsilon \tau_{\epsilon }$, all curves of Figure 5 merge into one curve showing that $\tau_{\epsilon }$ scales as 1/ϵ for a fixed *m*. Then, the numerical data suggests that $\tau_{\epsilon }=O\left(1/\epsilon \right)$ and in fact the maximal value of $\tau_{\epsilon }$ is approximately 0.8/ϵ. The same result is obtained if we take another localized initial state with its respective limiting distribution.

The fact that $\tau_{\epsilon }$ does not depend on *m* is impressive for several reasons. First, the diameter of the GBPG is O(m) and the classical mixing time is $O(m^{1.5}/\epsilon )$, both increase as a function of *m*. Second, the speed-up of the quantum mixing time over the classical mixing time is more than quadratic. Third, the authors of the seminal work in [5] suspected that quantum walks can mix at most quadratically faster than classical walks, but they were able to prove this result only for bounded degree graphs (see Section 7 of the work in [5]). Our numerical result is a strong evidence that the class of graphs GBPG is a counterexample of their conjecture.

**Figure 6:**
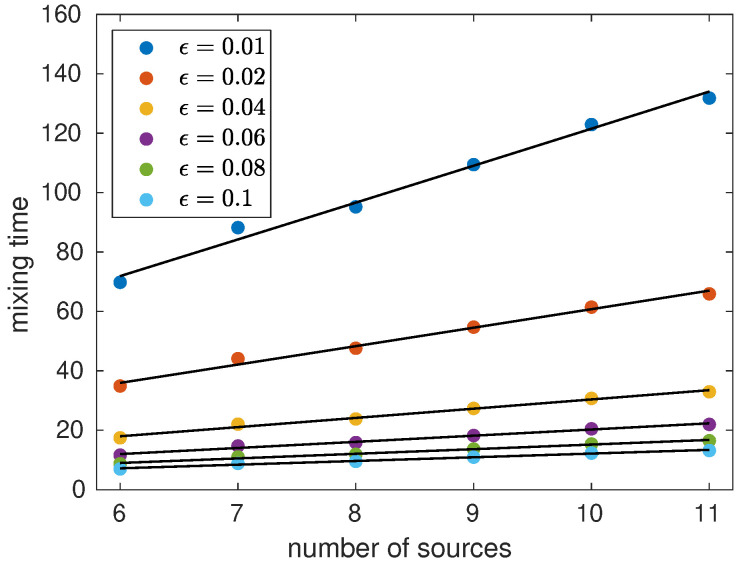
Quantum mixing time as a function of the number of sources *m* for ϵ from 0.01 to 0.1 using the alternative initial state (16).

To understand the key role that initial conditions play on the computational complexity of the mixing time, let us analyze the alternative initial state (16). Figure 6 depicts the quantum mixing time as a function of the number of sources *m* for ϵ from 0.01 to 0.1 for the initial state (16). To calculate the mixing time, we use the limiting distributions depicted in Figure 3. The numerical results show that $\tau_{\epsilon }\approx \left(0.12m+1.11\right)/\epsilon$. Note that in this case the mixing time increases when *m* increases, but the quantum walk still mixes faster than the classical random walk.

In order to better understand the different behaviors when we use these initial states, let us focus on the limiting distributions depicted in the right-hand panels of Figure 2 and Figure 3, which correspond to m=7. The probability at node 1 in the first case (Figure 2) is approximately 15% of the probability associated with the initial state at node 1. When we simulate the limiting distributions for *m* equal to 9 to 13, we note that the their overlap with the initial state increases, while the probability of the limiting distribution at the most distant node decreases. This means that the limiting distribution is close to the initial probability distribution. On the other hand, the probability at node 1 of the limiting distribution in the second case (Figure 3) decreases when *m* increases, meaning that the limiting distribution goes farther and farther away from the distribution associated with the initial state when *m* increases. The mixing time does not increase in the first case and must increase in the latter case. This analysis shows that the mixing time may have remarkably different behavior for some graph class that has very different limiting distributions depending on the choice of the initial state. That does not happen with classical random walks, whose mixing times are linearly lower bounded by the graph diameter.

**Figure 7:**
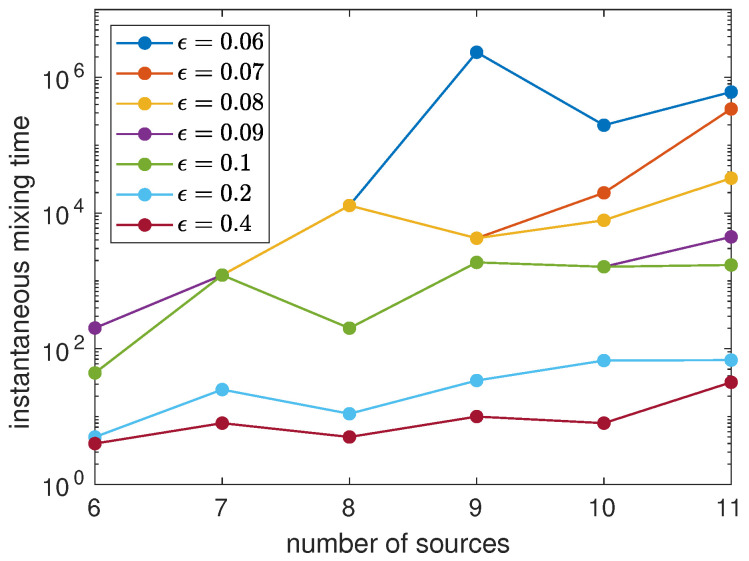
Instantaneous mixing time to the limiting distribution as a function of *m* for ϵ from 0.06 to 0.4 using the initial state (16).

Figure 7 depicts the instantaneous mixing time $I_{\epsilon }$ as a function of the number of sources *m* for ϵ from 0.06 to 0.4. The initial state is distributed on a maximal clique, both to obtain the limiting distribution and to calculate $I_{\epsilon }$. The figure suggests that $I_{\epsilon }$ increases exponentially as a function of *m*, which reinforces the notion that to sample efficiently from the limiting distribution we need to use the average probability distribution and then we need to implement the stochastic dynamics implied by the definitions involving averages.

## 6. Conclusions and Directions for Further Research

In this paper, we have analyzed the quantum mixing time of the coined quantum walk dynamics on a special class of TPG, GBPG, restricted to two consumers. The graphs in this class have the maximum number of nodes because the values of the capacities were modified by a perturbation method and one of the demands in TLP. To enhance our conclusions, we have also simulated the dynamics of a random walk on the same class of graphs to obtain the behavior of the classical mixing time. Using numerical experiments, we have showed that the classical mixing time increases in terms of the number of sources *m* as $O(m^{1.5})$.

Our numerical results suggest that the quantum walk provides a speedup in the time required for reaching the limiting distribution. With a localized initial state of the walk on a single node, we have found that the mixing time does not increase with the size of the graph, specified by the number of sources *m*. We have noticed that the limiting distribution in this case is close to the probability distribution of the localized initial state and in fact for larger values of *m* the distance between the limiting and the initial distributions decreases. Moreover, with a uniformly distributed initial state over a maximal clique of the graph, we observed that the mixing time increases as O(m), which is faster than the classical case. We have also analyzed the behavior of the instantaneous mixing time and found that the minimum time that the difference between the instantaneous probability distribution and the limiting distribution falls within a given error increases exponentially in terms of the number of sources.

The results of this paper provide promising steps in approaching the problems characterized on polytope graphs. In fact, an analytical approach to the structure of the GBPG provides a foundation for designing efficient quantum walk-based algorithms, which we intend to address. We also intend to address the asymmetry between parameters *m* and *n*, as we have noticed that the behavior of the mixing time as a function of *n* when we fix m=2 is different from the case we have analyzed.

## Figures and Tables

**Table 1:** Properties of the instances used in the computational tests.

Instance	*m*	*N*	Diam.	$1-\lambda_{1}$
1	2	2	1	2
2	3	6	3	1/2
3	4	12	3	1/3
4	5	30	5	1/4
5	6	60	5	1/5
6	7	140	7	1/6
7	8	280	7	1/7
8	9	630	9	1/8
9	10	1260	9	1/9
10	11	2772	11	1/10
11	12	5544	11	1/11
12	13	12,012	13	1/12
13	14	24,024	13	−
14	15	51,480	15	−

## Data Availability

No new data were created or analyzed in this study. Data sharing is not applicable to this article.
